# HIF-1α Regulates Glucocorticoid-Induced Osteoporosis Through PDK1/AKT/mTOR Signaling Pathway

**DOI:** 10.3389/fendo.2019.00922

**Published:** 2020-01-28

**Authors:** Wen-Ning Xu, Huo-Liang Zheng, Run-Ze Yang, Lei-Sheng Jiang, Sheng-Dan Jiang

**Affiliations:** Department of Clinic of Spine Center, Xinhua Hospital, Shanghai Jiaotong University School of Medicine, Shanghai, China

**Keywords:** glucocorticoid, HIF-1α, PDK1, osteoblast, osteoporosis

## Abstract

Long-term and high dose glucocorticoid treatment can cause decreased viability and function of osteoblasts, which leads to osteoporosis and osteonecrosis. In this study, we investigated the role and mechanism of action of HIF-1α in glucocorticoid-induced osteogenic inhibition in MC3T3-E1 cells. Our results showed that HIF-1α protein expression was reduced when MC3T3-E1 cells were exposed to dexamethasone (Dex) at varying concentrations ranging from 10^−9^ to 10^−6^ M. PDK1 expression was also decreased in MC3T3-E1 cells after dexamethasone treatment. MC3T3-E1 cells when treated with the glucocorticoid receptor antagonist RU486 along with dexamethasone showed enhanced HIF-1α expression. In addition, upregulated expression of HIF-1α was capable of promoting the osteogenic ability of MC3T3-E1 cells and PDK1 expression. However, the HIF-1α antagonist 2-methoxyestradiol (2-ME) had a reverse effect in MC3T3-E1 cells exposed to dexamethasone. Furthermore, the PDK1 antagonist dichloroacetate could repress the osteogenic ability of MC3T3-E1 cells, although HIF-1α was upregulated when transduced with adenovirus-HIF-1α construct. The PDK1 agonist PS48 was able to promote the osteogenic ability of MC3T3-E1 cells treated with dexamethasone. Importantly, the protein levels of p-AKT and p-mTOR were increased in MC3T3-E1 cells treated with dexamethasone after PS48 treatment. *in vivo*, the PDK1 agonist PS48 could maintain the bone mass of mice treated with dexamethasone. This study provides a new understanding of the mechanism of glucocorticoid-induced osteoporosis.

## Introduction

Glucocorticoids (GCs), as immunosuppressive and anti-inflammatory drugs, are used extensively to treat various disorders such as autoimmune and inflammatory diseases, among others (1, 2). Patients develop osteoporosis and osteonecrosis due to decreased viability and function of osteoblasts caused by long-term and high dose glucocorticoid treatment (3–5). GCs impair the survival and osteogenic ability of osteoblasts, which is believed to be the main physiopathologic mechanism of GC-induced bone loss (6). Recent studies have reported that apoptosis or autophagy of osteoblasts are involved in GC-induced osteogenic inhibition in bone cells (3, 7). Glucocorticoids destroy the bone and bone structure by controlling the differentiation of bone marrow-derived stem cells (BMSCs) which differentiate into adipocyte lineage but not osteoblast lineage in bone microenvironments (8). However, the mechanisms of glucocorticoid-induced osteogenic inhibition in osteoblasts remain unknown.

Hypoxia inducible factor-1α (HIF-1α) is highly expressed in hypoxic cells (9, 10). Recent studies have demonstrated that HIF-1α also plays an important role in cell survival in a normoxic environment (11–13). HIF-1α regulates target gene expression to control cell metabolism (14). The bone marrow microenvironment, which affects bone cells including osteoblasts and osteoclasts, and immune cells, is considerably hypoxic by nature (15). Mice with osteoblast-specific deletion of von Hippel-Lindau (VHL) and osteoblast-specific overexpression of HIF-1α show increased bone volume and osteoblast numbers (15, 16). HIF-1α is a transcription factor which regulates the levels of VEGF, resulting in high trabecular bone mass and increased bone vascular density (17–19). HIF-1α has also been reported to regulate PDK1 expression, but not VEGF, resulting in increased bone mass (15). Glucocorticoids suppressed the expression of HIF-1α and VEGF in osteoblasts and osteocytes of mice (20). However, the role of HIF-1α in glucocorticoid-induced osteogenic inhibition of osteoblasts is still unclear.

Pyruvate dehydrogenase kinase (PDK1) suppresses mitochondrial function through antagonizing the function of pyruvate dehydrogenase (PDH), a rate-limiting enzyme involved in the conversion of pyruvate to acetyl-coenzyme A, and entry into the tricarboxylic acid cycle (21). Moreover, PDK1 promotes cell proliferation via activating the AKT pathway (22, 23). Nevertheless, whether PDK1 inhibits osteoblast-induced osteoporosis by regulating glucocorticoid-induced osteoblasts is unclear. In this study, we investigated the role of HIF-1α and PDK1 in regulating glucocorticoid-induced osteogenic inhibition of osteoblasts.

## Materials and Methods

### Cell Culture and Treatments

The pre-osteoblast cell line MC3T3-E1 (subclone 4) was purchased from ATCC (USA) and cultured in α-MEM (#SH30265; Hyclone, GE Healthcare Life Sciences, Pittsburgh, PA, USA) containing 10% fetal bovine serum (FBS, #10099; Gibco, Thermo Fischer Scientific, Bartlesville, OK, USA) in 6 cm plates and incubated at 37°C with 5% CO_2_/95% air. Osteogenic differentiation was induced as described in a previous study (24). MC3T3-E1 cells were cultured in an osteogenic differentiation medium containing 4 mM glycerophosphate (#G9891; Sigma-Aldrich, St. Louis, MO, USA) and 25 μg/mL ascorbic acid (#A4403; Sigma-Aldrich) until 70% confluency. Dexamethasone (#D4902; Sigma-Aldrich, final concentration of ethanol, 0.01%, vol/vol) at different concentrations was then added to the osteogenic differentiation medium for 14 days. The culture medium was replaced every two days. MC3T3-E1 cells were cultured in a culture medium containing 10^−7^ M dexamethasone supplemented with or without RU486 (10^−5^ M), 2-ME (20 μM), DCA (10 mM), and PS48 (5 μM).

### Animals and Grouping

Fifteen male C57BL/6J mice (8 weeks of age, Shanghai SLAC Laboratory Animal Co., Ltd., Shanghai, China) were randomly assigned into three groups (*n* = 5 per group): control group (injected with empty adenoviral vector and intraperitoneal injection of normal saline), dexamethasone group (injected with empty adenoviral vector and intraperitoneal injection of dexamethasone), and dexamethasone + Ad-PDK1 group (intraperitoneal injection of dexamethasone and bone marrow injection of adenovirus carrying a promoter to overexpress PDK1). Dexamethasone (10 mg/kg bodyweight) was injected intraperitoneally for 21 days, as described in a previous study (25). All mice were euthanized. Bilateral femurs of mice were obtained for subsequent experiments. Male mice were used *in vivo* study in order to rule out the effects of estrogen. All animal care procedures were in accordance with the guidelines of the Ethics Committee of Xinhua Hospital Affiliated to Shanghai Jiao Tong University School of Medicine and were approved by this committee.

### Adenoviral Transduction

*In vitro*, MC3T3-E1 cells were cultured in six-well plates, and adenoviral solution (carrying empty vector or a promoter to overexpress HIF-1α or PDK1) was added into the growth medium. After 48 h, the medium containing adenovirus was removed and osteogenic differentiation medium was added. The target gene and protein expression were confirmed by qRT-PCR and western blotting, respectively. Adenoviral vectors were purchased from Hanbio Biotechnology Co., Ltd. (Shanghai, China).

*In vivo*, adenoviral particles carrying empty vector or a promoter to overexpress PDK1 were injected into the bone marrow cavity of bilateral femurs, at a dose of 20 μL virus solution (5 × 10^8^ pfu)/limb using a 25 μL microsyringe (Hamilton, 1702RN, Reno, NV, USA) every 2 weeks, as described in a previous study (26).

### ALP and Alizarin Red Staining Assay

MC3T3-E1 cells were washed with PBS thrice and fixed with 4% paraformaldehyde for 20 min after induction of osteogenic differentiation. ALP staining was performed for 30 min using the BCIP/NBT reagent kit (Leagene Biotechnology, Beijing, China). Alizarin red staining was performed using the Alizarin working solution (Cyagen Biosciences, Shanghai, China) for 5 min according to the manufacturer's instructions. After washing three times with PBS, the stained MEC3T3-E1 cells in each well were photographed. Each staining experiment was performed at least three times, separately.

### Western Blotting

Total protein was isolated from MC3T3-E1 cells using RIPA (Radio-Immunoprecipitation Assay, Beyotime, Shanghai, China) lysis buffer containing 1% PMSF (Phenylmethylsulfonyl fluoride, Beyotime, Shanghai, China). Protein samples were separated by SDS-PAGE and transferred onto polyvinylidene difluoride (PVDF) membranes. PVDF membranes containing proteins were blocked with 5% non-fat milk for 1 h and then incubated with specific primary antibodies overnight at 4°C. Primary antibodies included HIF-1α (1:1,000, Proteintech, USA), PDK1 (1:1,000, Proteintech, USA), Runx2 (1:1,000, Abcam, UK), OCN (1:1,000, Abcam, UK), ALP (1:1,000, Cell Signaling Technology, USA), total-Akt (1:2,000, Cell Signaling Technology, USA), phospho-Akt (1:1,000, Cell Signaling Technology, USA), total-mTOR (1:2,000, Cell Signaling Technology, USA), phospho-mTOR (1:1,000, Cell Signaling Technology, USA) and GAPDH (1:1,000, Beyotime, Shanghai, China). After washing three times with TBST for 5 min, the PVDF membranes were incubated with horseradish peroxidase-conjugated anti-rabbit secondary antibody for 1 h and target proteins were visualized using the Western Chemiluminescent HRP Substrate Kit (Millipore, Billerica, MA, USA).

### Hematoxylin-Eosin and Immunohistochemical Staining

The dissected femurs were fixed in 10% buffered formalin for 3 days, and then decalcified in 10% EDTA (pH = 7.0) at 4°C for 21 days, embedded in paraffin and sectioned for follow-up experiments. For hematoxylin-eosin staining, bone sections were stained with hematoxylin for 5 min and then stained with eosin for 2 min. For immunohistochemical staining, bone sections were incubated with PDK1-specific primary rabbit antibody (1:200 dilution) overnight at 4°C. Isotype control was incubated with rabbit IgG. A horseradish peroxidase-streptavidin detection system (Dako, Glostrup, Sweden) was used to examine the immunoreactivity.

### Micro-Computed Tomography Scanning and Quantitative Analysis

The femurs were fixed overnight in 70% ethanol and scanned using Scanco μCT 40 scanner (Scanco Medical AG, Zurich, Switzerland) at a resolution of 18 μm. Two/three dimensional images were obtained and analyzed to evaluate bone mass, bone mineral density (BMD), bone volume/total volume (BV/TV), trabecular thickness (Tb. Th), trabecular number (Tb. N), trabecular separation (Tb. Sp), Medullary area (Ma. Ar), Total area (Tt. Ar), Cortical bone thickness (Ct. Th), and Cortical Area (Ct. Ar) of the distal femur. We set the region of interest (ROI) as trabecular bone of 2-mm length below the epiphyseal growth plate.

### Statistical Analysis

All data are presented as the mean ± SD of three independent experiments and quantified relative to control experiments. The differences between multiple groups were analyzed by ANOVA and Tukey *post hoc* analysis. Differences were considered as statistically significant when p value was < 0.05. ***p < 0.001, **p < 0.01, *p < 0.05.

## Results

### Downregulation of HIF-1α and PDK1 by Dexamethasone

**Figure 1:**
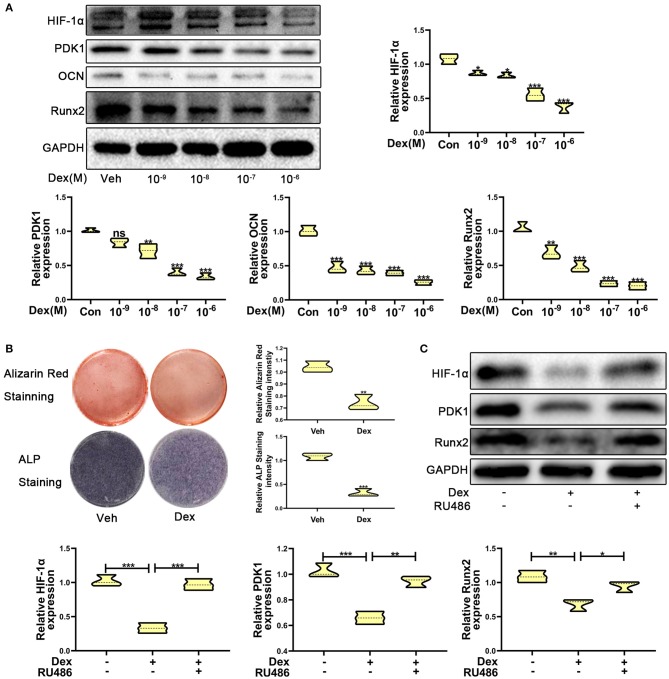
Dexamethasone inhibited the osteogenic function of MC3T3-E1 cells via regulating HIF-1α expression. **(A)** The protein expression levels of HIF-1α, PDK1, OCN, Runx2, and GAPDH were determined by western blotting in MC3T3-E1 cells treated with a varying concentration of dexamethasone for 14 days. **(B)** ALP and Alizarin red staining were performed to detect the osteogenic function of MC3T3-E1 cells treated with vehicle and dexamethasone. **(C)** Western blotting was performed to detect the protein expression levels of HIF-1α, PDK1, Runx2, and GAPDH in MC3T3-E1 cells treated with vehicle, dexamethasone, and dexamethasone + RU486, respectively. Differences were considered as statistically significant when *p* < 0.05. ***p < 0.001, **p < 0.01, *p < 0.05.

To investigate the expression of HIF-1α in glucocorticoid-induced osteogenic inhibition, MC3T3-E1 cells were exposed to dexamethasone at varying concentrations ranging from 10^−9^ to 10^−6^ M. The results of western blotting showed that the protein expression of HIF-1α was decreased (Figure 1A). The expression of osteogenic markers such as Runx2 and OCN were also decreased and these results confirmed that dexamethasone could repress the ability and function of osteoblasts. Recent reports have shown that HIF-1α could regulate PDK1 to mediate metabolic effects in different types of cells (15, 27). Therefore, we examined PDK1 expression by western blotting. As shown in Figure 1A, PDK1 expression was reduced in MC3T3-E1 cells exposed to dexamethasone. Based on results from a previous study (28) and our findings, we determined that 10^−6^ M of dexamethasone was the most optimal concentration. Treatment of MC3T3-E1 cells with 10^−6^ M dexamethasone for 7 days induced remarkable osteogenic inhibition (Figure 1B).

To further confirm whether dexamethasone treatment inhibits osteogenic ability of osteoblasts, MC3T3-E1 cells treated with dexamethasone were exposed to the glucocorticoid antagonist RU486 (10^−5^ M). As shown in Figure 1C, RU486 reversed the inhibitory effect of Dex to a similar level as observed in the control cells. These results revealed that dexamethasone inhibited osteogenic ability of osteoblasts through HIF-1α and PDK1. However, whether HIF-1α regulates the osteogenic inhibition induced by dexamethasone via PDK1 is still an unanswered question.

### Upregulation of HIF-1α Promotes Osteogenic Function Through Facilitating PDK1 Expression

**Figure 2:**
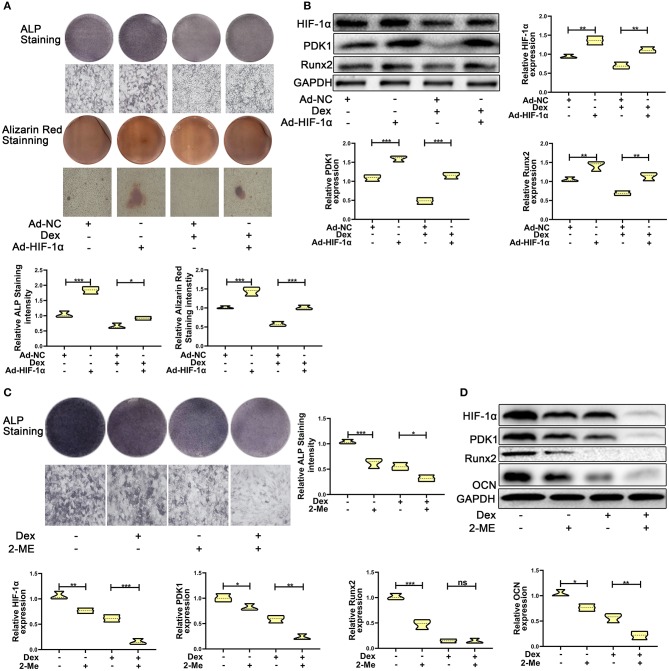
HIF-1α mediated dexamethasone-induced osteogenic inhibition of osteoblasts via regulating PDK1 expression. **(A)** Osteogenic ability was confirmed by ALP and Alizarin red staining in MC3T3-E1 cells treated with Ad-NC, Ad- HIF-1α + dexamethasone, Ad-NC + dexamethasone and Ad-HIF-1α + dexamethasone, respectively. Scale Bar = 20 μm. **(B)** The protein expression levels of HIF-1α, PDK1, Runx2, and GAPDH were detected by western blotting in MC3T3-E1 cells treated with Ad-NC, Ad-HIF-1α + dexamethasone, Ad-NC + dexamethasone and Ad-HIF-1α + dexamethasone, respectively. **(C)** Osteogenic ability was confirmed by ALP staining in MC3T3-E1 cells treated with empty vector, dexamethasone, 2-ME and dexamethasone + 2-ME, respectively. Scale Bar = 20 μm. **(D)** The protein expression levels of HIF-1α, PDK1, Runx2, OCN, and GAPDH were detected by western blotting in MC3T3-E1 cells treated with empty vector, dexamethasone, 2-ME and dexamethasone + 2-ME, respectively. Differences were considered as statistically significant when *p* < 0.05. ***p < 0.001, **p < 0.01, *p < 0.05.

To test whether overexpression of HIF-1α promotes dexamethasone-induced osteogenic inhibition in MC3T3-E1 cells, we overexpressed HIF-1α by transducing cells with adenoviral plasmid containing HIF-1α construct. As shown in Figure 2A, the results of ALP and Alizarin red staining showed that overexpression of HIF-1α led to increased osteogenic function in MC3T3-E1 cells. Western blotting results confirmed the above findings. The expression of the osteogenic marker Runx2 was increased. HIF-1α and PDK1 expression levels were also increased in MC3T3-E1 cells (Figure 2B).

To further affirm the role of HIF-1α in MC3T3-E1 cells exposed to dexamethasone, HIF-1α antagonist 2-methoxyestradiol (2-ME, 20 μM) was added to medium containing dexamethasone. The osteogenic inhibition induced by dexamethasone was enhanced in dexamethasone + 2-ME treated cells (Figure 2C). Inhibition of HIF-1α expression was also accelerated in dexamethasone + 2-ME treated cells. In addition, 2-ME enhanced the inhibition of PDK1 expression. 2-ME treatment showed increased inhibition of osteogenic markers Runx2 and OCN (Figure 2D). These results demonstrated that HIF-1α regulated dexamethasone-induced osteogenic inhibition via PDK1 expression.

### Repression of PDK1 Reverses the Protective Effect of HIF-1α on Glucocorticoid-Induced Osteogenic Inhibition

**Figure 3:**
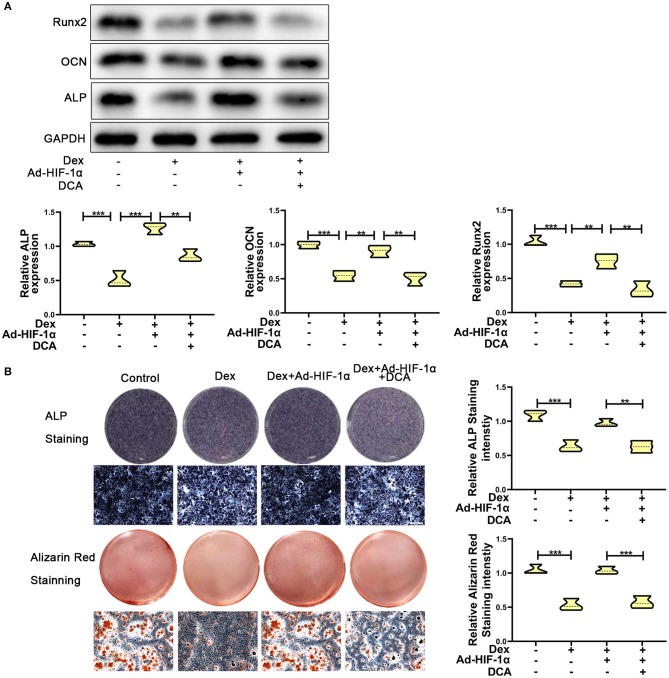
Repression of PDK1 reversed the protective effect of Adenovirus-HIF-1α on glucocorticoid-induced osteogenic inhibition in MC3T3-E1 cells. **(A)** The protein expression levels of osteogenic markers Runx2, OCN, ALP, and GAPDH were determined by western Blotting in MC3T3-E1 cells exposed to empty vector, dexamethasone, dexamethasone + Ad-HIF-1α, and dexamethasone + Ad-HIF-1α+ DCA, respectively. **(B)** ALP and Alizarin red Staining were performed to determine the osteogenic ability of MC3T3-E1 cells exposed to empty vector, dexamethasone, dexamethasone + Ad-HIF-1α, dexamethasone + Ad-HIF-1α+ DCA, respectively. Scale Bar = 20 μm. Differences were considered as statistically significant when *p* < 0.05. ***p < 0.001, **p < 0.01, *p < 0.05.

To further confirm whether HIF-1α regulates glucocorticoid-induced osteogenic inhibition via the PDK1 signaling pathway, PDK1 antagonist dichloroacetate (DCA, 10 mM) (29) was added to the medium. As shown in Figure 3A, western blotting showed that the expression levels of osteogenic markers such as Runx2, OCN, and ALP were increased in adenovirus- HIF-1α + dexamethasone group compared to adenovirus-NC + dexamethasone group. However, there was decreased expression of Runx2, OCN, and ALP in adenovirus + dexamethasone + DCA group compared to adenovirus-HIF-1α + dexamethasone group. The results of ALP and Alizarin red staining demonstrated that osteogenic ability of MC3T3-E1 cells was reduced in the adenovirus- HIF-1α + dexamethasone + DCA group compared to adenovirus-HIF-1α + dexamethasone group (Figure 3B). These results suggested that HIF-1α promoted the osteogenic ability of MC3T3-E1 cells treated with dexamethasone, by regulating PDK1 expression.

### Upregulation of PDK1 Facilitated the Osteogenic Ability of Osteoblasts After Glucocorticoid Treatment via the AKT/mTOR Pathway

**Figure 4:**
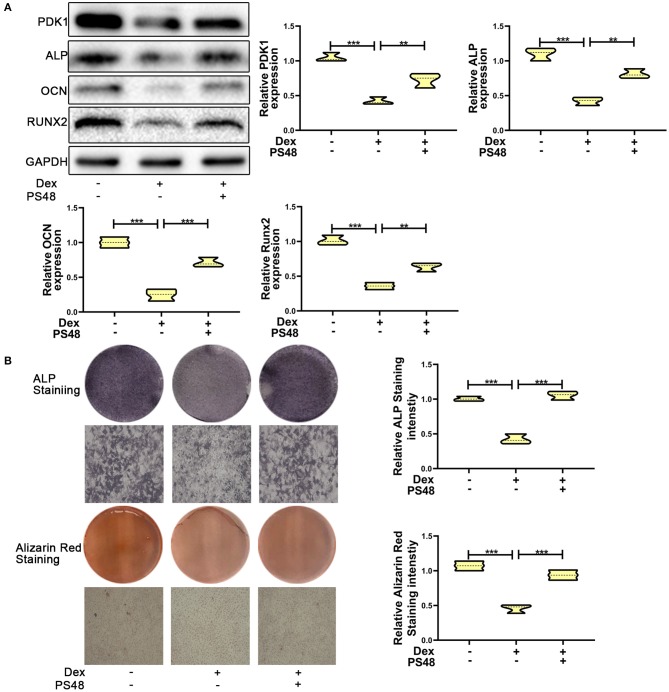
Upregulation of PDK1 promoted the osteogenic ability of MC3T3-E1 cells. **(A)** Western blotting was performed to detect the protein expression levels of PDK1 and osteogenic markers Runx2, OCN, ALP, PDK1, and GAPDH in MC3T3-E1 cells exposed to empty vector, dexamethasone, and dexamethasone + PS48, respectively. **(B)** ALP and Alizarin red staining were performed to determine the osteogenic ability of MC3T3-E1 cells exposed to empty vector, dexamethasone, and dexamethasone + PS48, respectively. Scale Bar = 20 μm. Differences were considered as statistically significant when *p* < 0.05. ***p < 0.001, **p < 0.01, *p < 0.05.

To verify the role of PDK1 in osteoblasts, we treated MC3T3-E1 cells with the PDK1 activator PS48 (5 μM) (30). PS48 promoted the expression levels of PDK1 in addition to osteogenic markers Runx2, OCN and ALP (Figure 4A). ALP and Alizarin red staining suggested that the osteogenic function of MC3T3-E1 cells treated with dexamethasone was enhanced by PS48 (Figure 4B). These results indicated that PDK1 was able to regulate glucocorticoid-induced osteogenic inhibition.

**Figure 5:**
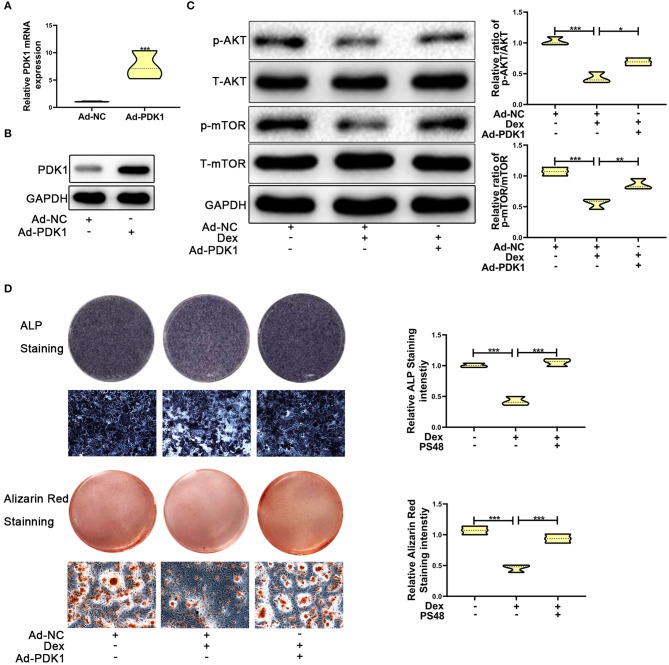
Upregulation of PDK1 by Adenovirus-PDK1 promoted the osteogenic ability of MC3T3-E1 cells by activating the AKT/mTOR pathway. **(A)** The expression of Adenovirus-PDK1 was confirmed by qRT-PCR. **(B)** The effect of Adenovirus-PDK1 was confirmed by qRT-PCR. **(C)** Western blotting was performed to detect the protein expression levels of p-AKT, total-AKT, p-mTOR, total-mTOR, and GAPDH in MC3T3-E1 cells exposed to empty vector, dexamethasone, and dexamethasone + Adenovirus-PDK1, respectively. **(D)** ALP and Alizarin red staining were performed to determine the osteogenic ability of MC3T3-E1 cells treated with empty vector, dexamethasone, and dexamethasone + Adenovirus-PDK1, respectively. Scale Bar = 20 μm. Differences were considered as statistically significant when *p* < 0.05. ***p < 0.001, **p < 0.01, *p < 0.05.

Several studies have reported that PDK1 regulates cellular metabolism by activating the AKT/mTOR pathway (30–32). Adenoviral -PDK1 construct or adenoviral -NC construct was transduced into MC3T3-E1 cells before exposure to dexamethasone. As shown in Figures 5A,B, the mRNA and protein expression of PDK1 was increased significantly in MC3T3-E1 cells after transduction with the adenoviral-PDK1 construct. Upregulation of PDK1 enhanced the ratio of p-AKT and p-mTOR in adenovirus-PDK1 + dexamethasone group compared to adenovirus-NC + dexamethasone group (Figure 5C). ALP and Alizarin red staining showed that upregulation of PDK1 could enhance the osteogenic ability of MC3T3-E1 cells (Figure 5D). These results revealed that PDK1 was able to regulate glucocorticoid-induced osteogenic inhibition of osteoblasts.

### Adenovirus-Mediated Delivery of PDK1 Increased the Bone Mass of Mice Treated With Glucocorticoid

**Figure 6:**
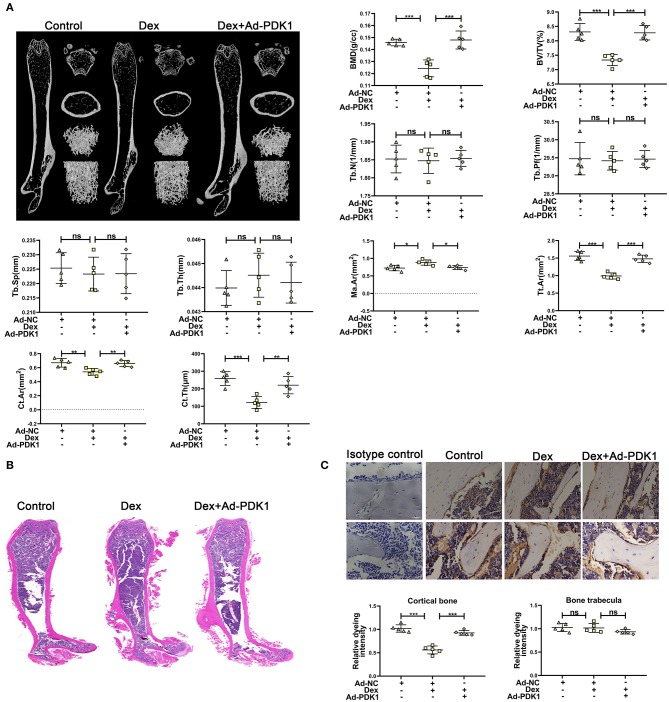
Upregulation of PDK1 improved the bone mass of mice treated with dexamethasone. **(A)** Representative μCT images and quantification analysis of bone parameters in femurs of control, dexamethasone and dexamethasone + Adenovirus-PDK1 mice. **(B)** Representative images of HE staining to examine the structure of femurs. **(C)** Immunohistochemical staining for detecting the protein expression of PDK1 in femurs of mice. Isotype control showed that the binding of the primary antibody was specific. Scale Bar = 5 μm. BMD, Bone Mineral Density; BV/TV, bone volume over total volume; Tb. Th, trabecular thickness; Tb. N, trabecular number; Tb. Sp, trabecular separation, Tt. Ar, Total area; Ma. Ar, Marrow area; Ct. Ar, Cortical area, Cortical bone thickness (Ct. Th) Differences were considered as statistically significant when *p* < 0.05. ***p < 0.001, **p < 0.01, *p < 0.05.

To confirm the protective effect of PDK1 *in vivo*, we evaluated the effect of adenovirus-mediated delivery of PDK1 (Ad-PDK1) in a mouse model of glucocorticoid-induced osteoporosis. When PDK1 was upregulated, the femur BMD and BV/TV in glucocorticoid-treated mice were significantly increased, as indicated by μCT analysis. However, there was no change in Tb. N, Tb. Pf, Tb. Sp, and Tb. Th (Figure 6A). Interestingly, marrow area (Ma. Ar) was increased after treatment with Dex and was reduced after upregulation of PDK1. Dex treatment reduced cortical bone thickness (Ct. Th), total area (Tt. Ar), and cortical area (Ct. Ar), but upregulation of PDK1 enhanced both these parameters. The femur trabecular number was not altered while the thickness of cortical bone was reduced. Hematoxylin-eosin staining of femur confirmed the results of μCT analysis (Figure 6B). PDK1 expression as determined by immunohistochemical staining was reduced in the cortical bone but not in the trabecular bone after treatment with dexamethasone (Figure 6C). Upregulation of PDK1 could improve the thickness of the cortical bone. We speculated that dexamethasone mainly affected the cortical bone. The mechanism of this phenomenon needs to be further studied.

## Discussion

In this study, we found that decreased HIF-1α expression was associated with dexamethasone-induced osteogenic suppression of osteoblasts in MC3T3-E1 cells. Excessive and long-term administration of glucocorticoids disturbed the osteogenic function of bone cells (3, 6). It has been reported that HIF-1α plays a positive role in the process of bone remodeling and osteoblast function (33–35). A recent study has demonstrated that HIF-1α was linked to glucocorticoid-induced osteoporosis in mice (20). HIF-1α is highly expressed in osteoblasts and osteocytes in a hypoxic environment and is involved in the process of bone metabolism (16, 36). However, the mechanism of HIF-1α in the regulation of glucocorticoid-induced osteogenic differentiation has not been studied adequately. Here, our results revealed for the first time that, the expression levels of HIF-1α and PDK1 were suppressed by dexamethasone treatment in MC3T3-E1 cells. Furthermore, HIF-1α regulated the negative effects of glucocorticoid on osteogenic function via the PDK1/AKT/mTOR signaling pathway.

MC3T3-E1 cells derived from C57/BL mouse calvaria are pre-osteoblasts possessing the ability to differentiate into osteoblasts and generate mineralized matrix in differentiation medium. Dexamethasone, a synthetic glucocorticoid, is used widely in clinical therapy, but excessive and long-term administration has a deleterious effect on bone formation. Increased concentration of dexamethasone (>10^−8^ M) significantly suppresses osteogenic differentiation and mineralization ability in osteoblasts (28). Inhibition of osteoblast proliferation and differentiation is considered to be the main reason for glucocorticoid-induced osteoporosis (4). However, the molecular mechanisms of glucocorticoid-induced osteogenic inhibition of osteoblasts are still unclear.

A previous study has demonstrated that HIF-1α regulated bone formation via improving VEGF-A levels (33). Glucocorticoids could suppress HIF-1α protein expression (20). In this study, the expression of HIF-1α was decreased in MC3T3-E1 cells treated with different concentrations of dexamethasone. The administration of glucocorticoid receptor (GR) blocker RU486 promoted osteogenic ability and decreased the expression of HIF-1α in MC3T3-E1 cells after dexamethasone exposure. These results demonstrated that glucocorticoid inhibited osteogenic function via the glucocorticoid receptor (28, 37). Upregulation of HIF-1α by transduction with adenoviral- HIF-1α construct could inhibit the deleterious effects of dexamethasone on osteogenic function in MC3T3-E1 cells, while downregulation of HIF-1α by antagonist 2-ME enhanced the negative effects of dexamethasone. Importantly, upregulation of HIF-1α could improve the decreased expression of PDK1, while suppression of HIF-1α accelerated decreased PDK1 expression. These results reveal that HIF-1α might antagonize glucocorticoid-induced osteogenic inhibition of osteoblasts via PDK1 signaling pathway.

To further confirm the mechanism by which PDK1 counteracts glucocorticoid-induced inhibition of osteogenesis, MC3T3-E1 cells transduced with adenoviral-HIF-1α construct were cultured in differentiation medium containing dexamethasone and DCA. DCA could reverse the protective effects of HIF-1α on glucocorticoid-induced osteogenic inhibition of osteoblasts. Moreover, the PDK1 activator PS48 was capable of repressing the deleterious effects of glucocorticoid treatment. These results proved that HIF-1α reduced glucocorticoid-induced osteogenic inhibition of osteoblasts via the PDK1 signaling pathway (15).

To explore the specific molecular mechanisms by which PDK1 reversed glucocorticoid-induced osteogenic inhibition, we investigated the response of AKT/mTOR signaling pathway. Recent studies have demonstrated that the AKT/mTOR signaling pathway regulates various biological activities in osteoblasts (38, 39). Hypoxia was reported to induce osteogenic differentiation via ILK, AKT, mTOR, and HIF-1α pathways (19). In addition, PDK1 has been reported to regulate various cellular metabolic processes via the AKT/mTOR signaling pathway (30, 40, 41). Therefore, we presumed that PDK1 reversed the glucocorticoid-induced osteogenic inhibition of osteoblasts via the AKT/mTOR signaling pathway. In this study, PDK1 adenoviral construct was transduced into MC3T3-E1 cells and the results showed that upregulation of PDK1 promoted the osteogenic function of osteoblasts. To further confirm the effect of PDK1 *in vivo*, PDK1 adenovirus was injected into the bone marrow cavity of femurs of mice. Overexpression of PDK1 reduced the marrow area (Ma. Ar) and increased the cortical bone thickness (Ct. Th), total area (Tt. Ar) and cortical area (CT. Ar). Upregulation of PDK1 significantly promoted the bone mass of mice treated with dexamethasone. The above results revealed that HIF-1α/PDK1 axis reversed the glucocorticoid-induced osteogenic inhibition of osteoblasts via activating the AKT/mTOR signaling pathway.

Our study presents two questions. First, although it has been reported that HIF-1α could mediate the osteogenic ability of osteoblasts, and improve bone mass (33), the role of VEGF-A in glucocorticoid-induced osteogenic inhibition of osteoblasts has not been confirmed yet. Second, whether PDK1 mediates glucocorticoid-induced osteogenic inhibition of osteoblasts via regulating mitochondrial metabolism remains to be studied. Future studies are required to resolve these questions.

In conclusion, we demonstrated that HIF-1α antagonized glucocorticoid-induced osteogenic suppression of osteoblasts via the PDK1/AKT/mTOR signaling pathway. Our findings provide novel insights to further understand the molecular mechanisms of glucocorticoid-induced osteogenic inhibition of osteoblasts. Specific overexpression of PDK1 could be an alternative method to alleviate glucocorticoid-induced osteoporosis.

## Data Availability Statement

Publicly available datasets were analyzed in this study. This data can be found here: winningxu@sjtu.edu.cn.

## Ethics Statement

The animal study was reviewed and approved by Ethics Committee of Xinhua Hospital Affiliated to Shanghai Jiao Tong University School of Medicine.

## Author Contributions

W-NX, S-DJ, and L-SJ conceived and designed the experiments. W-NX and H-LZ performed the experiments. W-NX and R-ZY acquired and analyzed the data. W-NX drafted the manuscript. S-DJ and L-SJ helped perform the analysis with constructive discussions and revised the manuscript.

### Conflict of Interest

The authors declare that the research was conducted in the absence of any commercial or financial relationships that could be construed as a potential conflict of interest. The reviewer HQ declared a shared affiliation, with no collaboration, with the authors to the handling editor at the time of the review.
